# Multidimensional analysis of floral scent emission patterns in *Phalaenopsis* ‘Chanel’

**DOI:** 10.1186/s12870-026-08738-w

**Published:** 2026-04-25

**Authors:** Ling Ding, Li Xu, Kejing Feng, Jiwei Ruan, Lifang Wu, Chunmei Yang, Rongpei Yu

**Affiliations:** 1 Flower Research Institute, Yunnan Academy of Agricultural Sciences, National Engineering Research Center for Ornamental Horticulture, Key Laboratory for Flower Breeding of Yunnan Province, Kunming, Yunnan 650205 China; 2 Yuxi Yunxing Biotech Co., Ltd, Yuxi, Yunnan 653100 China; 3 State Key Laboratory of Green Pesticide, Center for R&D of Fine Chemicals of Guizhou University, Guiyang, 550025 China; 4 Resource Plant Research Institute, Yunnan University, Kunming, Yunnan 650604 China

**Keywords:** *Phalaenopsis*, Floral scent, Volatile organic compounds (VOCs), Circadian rhythm

## Abstract

*Phalaenopsis* spp. is known as the best-selling potted flower in the world, while only a few cultivars possess floral scent, and the analysis of floral scent emission is limited. In present study, *Phalaenopsis* ‘Chanel’ with sweet and pleasant floral scent was used to explore the floral scent emission pattern by gas chromatography-mass spectrometry (GC-MS). A total of 21 VOCs were detected in blooming flowers, among which monoterpene linalool with an exceptional odor activity value of 1443.5 was identified as the vital VOC determining the aromatic profile of *Phal.* ‘Chanel’. The scent emission of *Phal.* ‘Chanel’ was multi-dimensionally regulated by spatial distribution and temporal dynamics. The scent emission pattern in spatial distribution was that the highest concentrations of both total VOCs and linalool were localized within the labellum, identifying it as the most fragrant floral structure. In the temporal dynamic pattern, the scent emission varied with floral development and circadian rhythm, and peaked in the bloom stage, especially on the 5th day after bloom (DAB) between 9:00 am and 11:00 am. Overall, the floral scent emission in *Phal.* ‘Chanel’ was dominated by linalool, and exhibited the spatiotemporal dynamics.

**Supplementary Information:**

The online version contains supplementary material available at 10.1186/s12870-026-08738-w.

## Introduction

*Phalaenopsis* spp. belonging to the Orchidaceae family, is known as the best-selling potted flower in the global market because of the captivating flower shape, diverse flower colors and long flowering period [1, 2]. Although some native species of *Phalaenopsis* have floral scent, the genetic stability of aromatic profile is degenerated during artificial crossbreeding, which leads to most cultivars of *Phalaenopsis* lacking floral scent, and only a limited number of cultivars exhibit detectable aromatic profile [1].

Floral scent is an important ornamental trait of plants and also plays an important role in pollination biology [3, 4]. Actually, floral scent is a mixture of volatile organic compounds (VOCs) with the low molecular weights (30 ~ 300 amu) [5]. To date, over 1700 VOCs have been identified in the flowers of ornamental plants. Interestingly, they are derived from only a few biochemical networks, which include the terpenoid, phenylpropanoid/benzenoid and fatty acid biosynthetic pathways [6].

The composition and concentration of floral VOCs vary significantly among different species as well as different cultivars in the same species, resulting in distinct aromatic profiles [6, 7]. Additionally, the composition and concentration of floral VOCs also fluctuate in different floral developmental stages, different floral structures, and the circadian rhythms, all of which contribute to dynamic changes in aromatic profile [8–10]. Notably, floral scent is an important signal substance for angiosperms to attract pollinators [11]. The emission time of floral scent is often synchronized with the circadian rhythms of target pollinators, thereby enhancing pollination efficiency. This temporal alignment represents an adaptive evolutionary mechanism that promotes successful plant-pollinator interactions [12, 13].

In ornamental plants, the floral scent profile of some species, such as *Rosa Damascena* [14], *Jasminum sambac* [15], and *Lilium* spp [16]. had been extensively studied. Currently, the floral VOCs of scented *Phalaenopsis* were only investigated in several native species and cultivars. In the scented native species *Phal. bellina* and *Phal. violacea*, the major floral VOCs were identified as linalool, geraniol and their derivatives [1, 8]. In the scented cultivars *Phal.* ‘Peter’s Pride’, *Phal.* ‘Cherry Tomatos’, *Phal.* ‘Tzu Chiang Balm’, and *Phal.* ‘Purple Butterfly’, a total number of 17 key VOCs were identified as major contributors to their floral aromatic profile based on odor activity values [17]. So far, there are limited reports on the multidimensional analysis of the floral scent emission of *Phalaenopsis*.

In the present study, *Phalaenopsis* ‘Chanel’ with a pleasant scent was used as the material to detect floral VOCs by headspace solid-phase microextraction combined with gas chromatography-mass spectrometry (HS-SPME/GC-MS). Furthermore, the scent emission patterns in different floral structures, different floral developmental stages as well as a 24-hour period were also investigated in *Phalaenopsis* ‘Chanel’. Our study will be beneficial to identify the key floral VOCs and floral scent emission patterns of scented *Phalaenopsis*.

## Materials and methods

### Plant materials and sampling

**Fig. 1:**
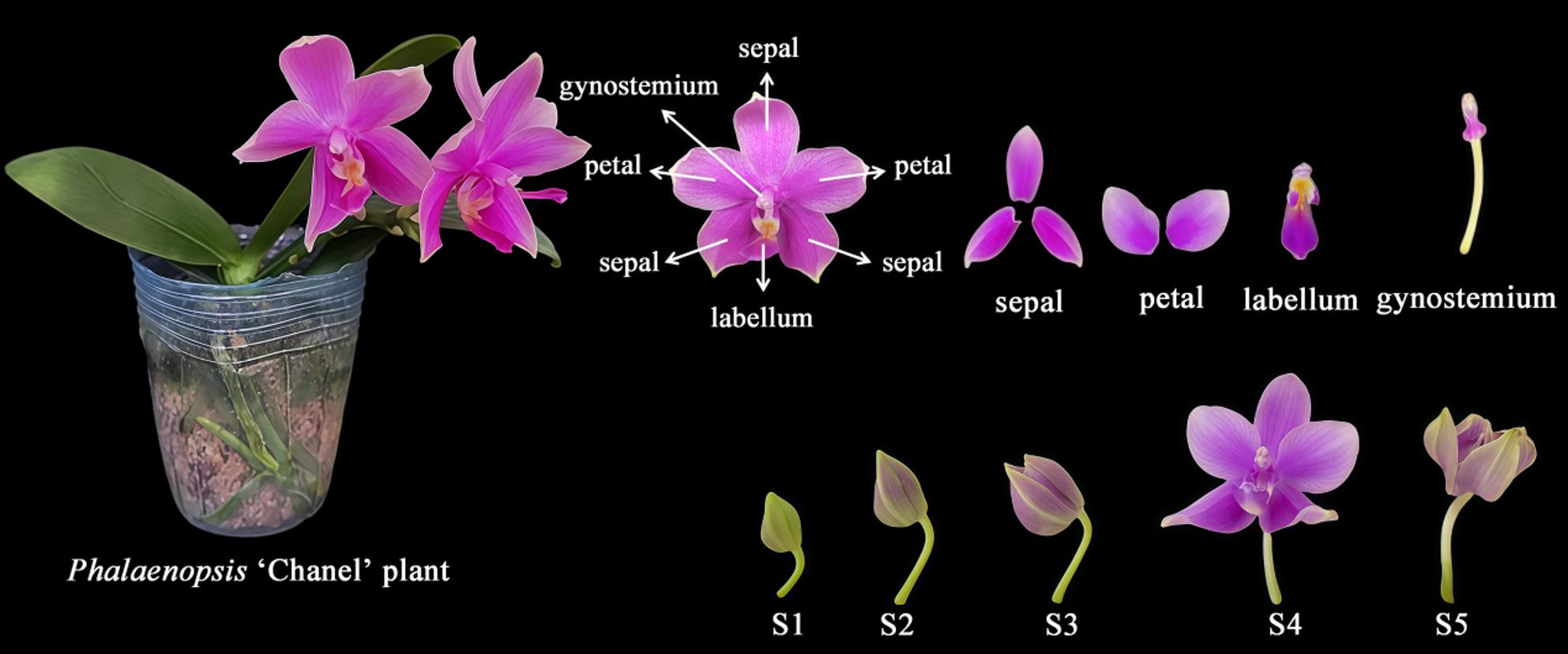
The plant, four floral structures and five floral developmental stages of the scented *Phalaenopsis* ‘Chanel’

The scented *Phalaenopsis* ‘Chanel’ were planted in the greenhouse of Flower Research Institute, Yunnan Academy of Agricultural Sciences (Jiangchuan District, Yuxi City, Yunnan province, China). The plants of *Phalaenopsis* ‘Chanel’ in the bloom stage (Fig. 1) were transferred to the phytotron. The photoperiod of phytotron was 8-hour dark and 16-hour light (6:00–22:00), and the relative humidity and temperature were 40 ± 2% and 25 ± 1 ℃, respectively.

The flowers on the 5th day after bloom (DAB) were used to detect and identify the floral VOC emission of blooming flower. Four floral structures, i.e., sepals, petals, labellum, and gynostemium (Fig. 1) dissected from the flowers on the 5th DAB were used to investigate the scent emission of different floral structures. Flowers from five developmental stages (S1 ~ S5, Fig. 1), including floral bud stages (S1 ~ S3), bloom stage (S4) and withering stage (S5), were used as materials to detect scent emission of different floral developmental stages (Fig. 1). The flowers on the 1st day after bloom (1 DAB) were selected to detect VOCs from the 1st day after bloom (1st DAB) to the 10th day after bloom (10 DAB) for dynamic monitoring of scent emission in different blooming days. Moreover, the flowers on the 5th DAB were used to explore the circadian rhythm of floral scent emission by detecting VOCs every two hours in a 24-hour period. Three independent biological replicates were used for all detection of scent emission.

### HS-SPME extraction

HS-SPME extraction was conducted by using a manual SPME injector (Supelco, Bellefonte, PA, USA), which was equipped with a 75 μm polydimethylsiloxane SPME fiber. Before extraction, the fiber was thermally conditioned at 250 ℃ for 3 min in GC-MS system (Trace GC Ultra/ITQ 900, Thermo Fisher Scientific, Waltham, MA, USA).

Based on different detection purposes, two methods of HS-SPME extraction, i.e., in vitro extraction and in vivo extraction, were adopted in the present study. In vitro extraction used the flowers or floral structures detached from plants as materials, while in vivo extraction used the flowers on the plants as materials, allowing the flowers to continue growing normally. In vitro extraction, which provided the sufficient adsorption for VOCs, was commonly used to detect and identify the all VOCs in the flowers [17]. In vivo extraction was mainly applied in the dynamic monitoring the vital VOCs of the same flower [10]. The apparatus for in vitro and in vivo extraction without samples were used as the blank controls, and their VOCs were analyzed for the background (Supplementary data 1).

In order to detect and identify the all VOCs of blooming flowers, different floral structures and different developmental flowers, the floral VOCs of these samples detached from plants were collected by in vitro extraction at 9:00 am. After recording the fresh weight (FW, g), the sample was immediately put into 20 ml SPME bottle with a 20 mm opening sealed using PTFE/silicone septum and an aluminum gland (Thermo Fisher Scientific, Waltham, MA, USA). Then 1 µl ethyl decanoate (CAS 000110-38-3) with a concentration of 1‰ (v/v) was added to the SPME bottle as an internal standard substance. The bottle was promptly capped, and the SPME fiber was inserted into the capped vial with the fiber positioned 1 cm above the flower. The adsorption of floral VOCs took place at the temperature of 30 ℃ for 30 min.

For dynamic monitoring VOCs in different blooming days, VOCs of the same flower on the plant were collected by in vivo extraction at 9:00 am from the 1st DAB to 10th DAB. Moreover, VOCs of the same flower on the 5th DAB were collected by in vivo extraction on the plant every two hours in a 24-hour period to investigate the VOC variation along with circadian rhythm. For in vivo extraction, the flower on the plant was covered with the collection bottle sealed with parafilm, then SPME injector was inserted through parafilm with SPME fiber positioned 1 cm above the flower. The adsorption of floral VOCs took place in the phytotron at temperature of 25 ± 1 ℃ for 30 min. After the last dynamic monitoring, the flower was detached from the plant, its fresh weight (FW, g) was recorded .

### GC-MS analysis

Once HS-SPME extraction process was completed, the SPME fiber was transferred to GC-MS injection port for desorption at 250 °C for 1 min. Subsequently, GC-MS was employed to collect data. In the gas chromatography, a capillary column of HP-5MS (30 m × 250 μm × 0.25 μm, Agilent J&W, Santa Clara, CA, USA) was utilized, with helium (99.999%) serving as the carrier gas at a flow rate of 1.0 mL/min. The split ratio was set at 10:1. The heating program and mass spectrometry were executed according to the parameters described by Song et al. [17].

### VOC identification

All VOCs (initial threshold of peak ≥ 19.5) were identified by comparing retention times (RT) and mass fragmentation patterns with the NIST library (National Institute of Standards and Technology 2011, Shimadzu, Japan). After that, the retention indices (RI) of VOCs were calculated relative to the n-alkanes (C6–C40) and compared to those of VOCs in the NIST online database (https://webbook.nist.gov/chemistry/cas-ser.html) to ensure the accuracy of VOC identification. VOCs of the blank controls (Supplementary data 1) and non-biosynthetic VOC were removed from the VOC lists of all samples. Moreover, the vital VOC identification was examined by matching the GC-MS result of the standard substance (Supplementary data 2).

### Calculation of VOC concentration and odor activity value

The calculation method of VOC concentration varied with different HS-SPME extraction methods, i.e., in vitro extraction and in vivo extraction. For in vitro extraction, ethyl decanoate was use as an internal standard, and the concentration of each VOC was calculated using the following formula:

$$\:\text{VOC concentration}(\mathrm{ng}\cdot\mathrm{g}\,\mathrm{FW}^{-1})=\frac{\text{Peak area of VOC}}{\text{Peak area of internal standard}}=\frac{\text{Concentration of internal standard}\,(\mathrm{ng}\cdot\mu\mathrm{L}^{-1})\times \text{Volume of internal standard}\ (\mu\mathrm{L})} {\text{Fresh weight of sample}\,(\mathrm{g})}$$

For in vivo extraction, linalool was selected as the standard substance and gradient diluted with n-hexane to check VOC identification and obtain the standard curve (Supplementary data 2). The content of each VOC was calculated by the standard curve *y* = 4.97 × 10^7^*x* -1.85 × 10^8^ (R^2^ = 0.9992), where *y* represents the peak area and *x* represents the content of VOC (ng). VOC concentration (ng·g^− 1^) was calculated by following formula:

$$\:\text{VOC concentration}(\mathrm{ng}\cdot\mathrm{g}\,\mathrm{FW}^{-1})=\frac{\text{VOC content}\,(\mathrm{ng})}{\text{Fresh weight of sample}\,(\mathrm{g})}$$

The odor activity value (OAV) is an important indicator for evaluating the contribution of VOC to floral scent, which is determined by the concentration and odor threshold of each VOC. Only VOCs with OAV>1 were identified as the key VOCs for floral scent [18]. The odor descriptions of VOCs and odor thresholds in water were mainly cited from Gemert [19], and the Volatile Compounds in Food online database (http://www.vcf-online.nl/Vcf Home.cfm). OAV was calculated using the following formula [18]:

$$\:\mathrm{O}\mathrm{A}\mathrm{V}=\frac{\text{VOC concentration} (\mathrm{ng}\cdot\mathrm{g}\text{ FW}^{-1})}{\text{VOC odor threshold}(\mathrm{ng}\cdot\mathrm{g}^{-1})}$$

### Data analysis

All data were analyzed by using Microsoft Excel 2021. Origin2021 and TBtools were employed to plot. Aroma types was obtained from ‘The Good Scents’ company web database (www.thegoodscentscompany.com).

## Results

### VOC detection and key VOC identification of blooming flowers

**Fig. 2:**
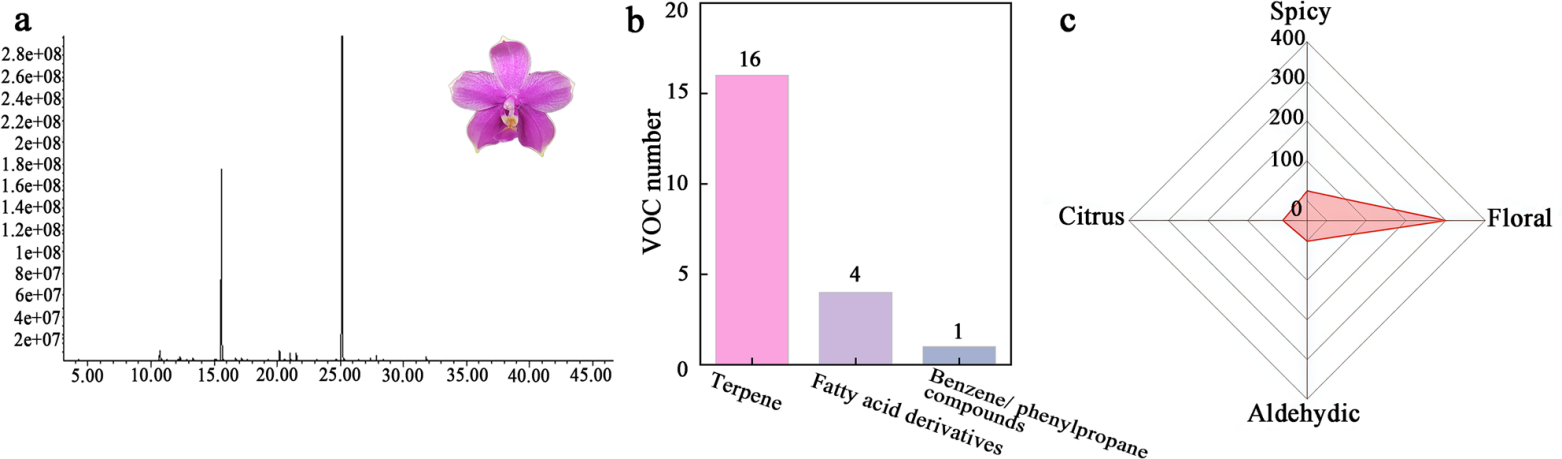
VOC detection and analysis of blooming flowers in *Phalaenopsis* ‘Chanel’ by in vitro extraction. **A** GC-MS total ion chromatograms. **B** VOC classification. **C** Radar chart of aroma types for the five key VOCs

Volatile organic compound (VOCs) of blooming flowers in *Phal.* ‘Chanel’ were detected by HS-SPME/GC-MS (Fig. 2a). 21 VOCs were identified (Table 1), most of which belong to terpenoids (Fig. 2b). Among all VOCs, the monoterpene linalool exhibited the highest concentration at 288.7 ± 6.76 ng·g FW^− 1^, followed by myrcene at 24.39 ± 1.93 ng·g FW^− 1^. Considering linalool with the highest concentration in *Phal.* ‘Chanel’, the standard substance of linalool was introduced to check VOC identification. The VOC identification of linalool studied here was confirmed to be correct by matching the GC-MS result of the standard substance (Supplementary data 2).

**Table 1:** The concentrations and odor activity values (OAVs) of volatile organic compounds (VOCs) in the blooming flowers of *Phalaenopsis* ‘Chanel’ by in vitro extraction

NO.	Family	CAS	VOC	RT(min)	RI		VOC concentration (ng·g^− 1^)	OT (ng·g^− 1^)	OAV
					HP-5	NIST			
1	Terpene	000078-70-6	Linalool	15.6017	1098	1099	288.7 ± 6.76	0.20	1443.5
2		000123-35-3	Myrcene	10.6371	982	991	24.39 ± 1.93	1.20	20.3250
3		000106-24-1	Geraniol	21.0246	1256	1255	11.64 ± 0.52	1.00	11.6400
4		005392-40-5	Citral	21.5203	1272	1270	10.86 ± 0.56	0.50	21.7200
5		000099-86-5	Alpha-Terpinene	11.7782	1010	1017	0.50 ± 0.12	2140.00	0.0002
6		000527-84-4	o-Cymene	12.1517	1018	1022	2.40 ± 0.79	24.10	0.0996
7		005989-27-5	(4R)-Limonene	12.3247	1022	1030	9.56 ± 1.03	34.00	0.2812
8		013877-91-3	β-Ocimene	12.8695	1035	1037	2.10 ± 0.32	34.00	0.0618
9		000502-99-8	α-Ocimene	13.3353	1046	1047	4.39 ± 0.76	-	-
10		000099-85-4	γ-Terpinene	13.7412	1055	1060	0.65 ± 0.18	-	-
11		007216-56-0	Neo-alloocimene, stab	16.9528	1135	1131	5.57 ± 0.32	-	-
12		000106-25-2	Nerol	20.1930	1229	1228	15.19 ± 0.61	53.00	0.2866
13		000106-26-3	Neral	20.5947	1242	1240	1.91 ± 0.28	53.00	0.0360
14		013474-59-4	Trans-α-Bergamotene	26.1870	1439	1435	0.28 ± 0.08	-	-
15		000644-30-4	α-Curcumene	27.2309	1480	1483	3.24 ± 0.75	-	-
16		073209-42-4	trans-Calamenene	28.4077	1527	1529	0.44 ± 0.11	-	-
17	Fatty acid derivatives	000124-19-6	Nonanal	15.7629	1102	1104	1.37 ± 0.19	1.10	1.2455
18		000928-96-1	Leaf alcohol	5.3502	831	857	0.70 ± 0.19	13.00	0.0538
19		010340-23-5	Cis-3-Nonen-1-ol	17.6521	1155	1156	1.25 ± 0.28	85.00	0.0147
20		000112-12-9	Undecan-2-one	22.2306	1294	1294	0.44 ± 0.07	18.20	0.0242
21	Benzene/phenylpropane	001461-02-5	Dihydrocurcumene	26.4553	1449	1448	0.82 ± 0.15	-	-

However, the contributions of VOCs to the olfactory scent cannot be determined solely by VOCs concentrations, because human olfactory perception also depends on VOC odor threshold. Therefore, odor activity value (OAV) of VOCs were calculated to identify the key VOCs responsible for the floral scent of *Phal.* ‘Chanel’. Among 21 VOCs in *Phal.* ‘Chanel’ flowers, 6 VOCs could not be assigned OAVs because their odor thresholds were absent in the literature (Table 1). In the other 15 VOCs, five VOCs (OAV >1) were identified as the key VOCs of the floral scent in *Phal.* ‘Chanel’ (Tables 1 and 2). Given that OAV of linalool (1443.5) was markedly higher than those of other key VOCs (1.25 ~ 21.72), linalool was identified as the vital VOC for floral scent of *Phal.* ‘Chanel’.

**Table 2:** The odor activity values (OAVs) and aroma types of five key volatile organic compounds (VOCs) in the blooming flowers of *Phalaenopsis* ‘Chanel’

VOC	VOC concentration (ng·g^− 1^)	OAV	Odor type
Myrcene	24.39 ± 1.93	20.33	Spicy
Linalool	288.70 ± 6.76	1443.5	Floral
Geraniol	11.64 ± 0.52	11.64	Floral
Nonanal	1.37 ± 0.19	1.25	Aldehydic
Citral	10.86 ± 0.56	21.72	Citrus

To further explore the sensory attributes of the floral scent of *Phal.* ‘Chanel’, the five key VOCs were classified according to their aroma types (Table 2), and the radar map was constructed to visualize their contributions (Fig. 2c). The results showed that linalool and geraniol were categorized as floral-type compounds, which played a dominant role in floral scent, defining the sweet and pleasant scent of *Phal.* ‘Chanel’.

### VOC detection of different floral structures

**Fig. 3:**
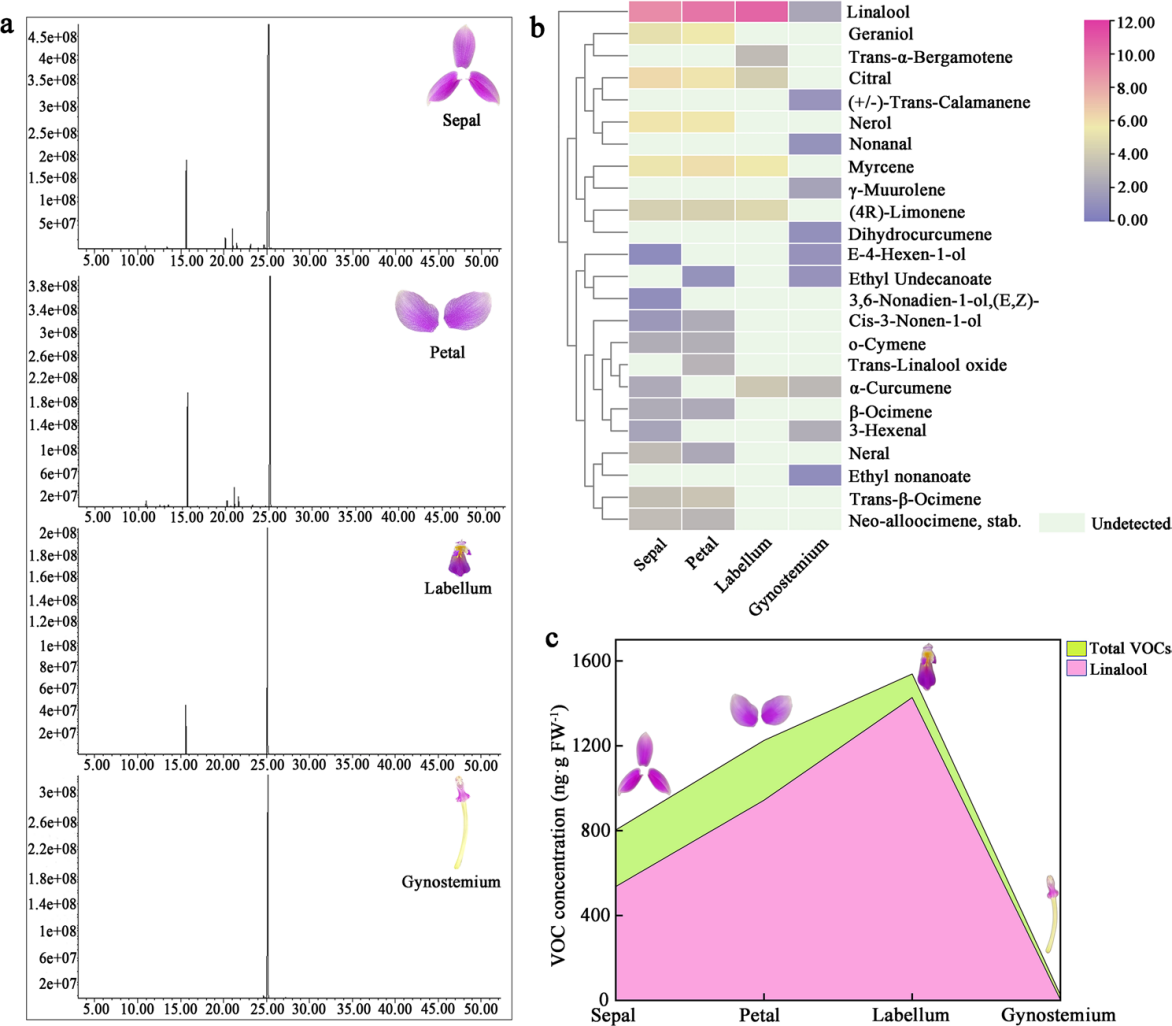
VOC detection and analysis of different floral structures in *Phalaenopsis* ‘Chanel’ by in vitro extraction. **A** GC-MS total ion chromatograms. **B** Heat map of VOC concentrations. Color bar represents the normalized VOC concentrations, warmer color indicates higher VOC emission, while cool color indicates lower VOC emission. **C** Concentration area map of total VOC and the vital VOC linalool

*Phalaenopsis* flowers possess a unique floral morphology comprising four distinct floral structures: sepals, petals, labellum, and gynostemium (Fig. 1). These structures differ in both morphology and function. In order to explore the spatial distribution of floral scent emissions, VOCs of four floral structures above were detected (Fig. 3a; Table 3). The results revealed that the number of VOCs varied among the floral structures. The sepals (17 VOCs) and petals (15 VOCs) exhibited relatively diverse VOCs, while the labellum (6 VOCs) released a limited VOCs (Fig. 3b). As for the five key VOCs identified above, only linalool was detected in all four floral structures (Fig. 3b).

**Table 3:** The concentrations of volatile organic compounds (VOCs) in different floral structures of *Phalaenopsis* ‘Chanel’ by in vitro extraction

NO.	Family	CAS	VOCs	RT(min)	RI		VOC concentration (ng·g^− 1^)			
					HP-5	NIST	Sepal	Petal	Labellum	Gynostemium
1	Terpene	000078-70-6	Linalool	15.6558	1100	1099	536.53 ± 74.91	943.56 ± 99.96	1427.49 ± 165.54	3.48 ± 0.83
2		000123-35-3	Myrcene	10.8646	987	991	41.91 ± 6.5	64.06 ± 2.8	46.37 ± 0.18	-
3		000106-24-1	Geraniol	21.0250	1256	1255	33.64 ± 1.54	47.95 ± 0.94	-	-
4		005392-40-5	Citral	21.5207	1272	1270	73.56 ± 11.43	55.68 ± 4.69	17.42 ± 1.84	-
5		000106-25-2	Nerol	20.1908	1229	1228	53.5 ± 2.93	51.33 ± 1.45	-	-
6		000106-26-3	Neral	20.5962	1242	1240	8.39 ± 0.89	3.72 ± 1.38	-	-
7		005989-27-5	(4R)-Limonene	12.4465	1025	1031	18.53 ± 2.23	19.03 ± 3.19	25.44 ± 1.46	-
8		003779-61-1	Trans-β-Ocimene	13.4201	1048	1049	9.27 ± 1.69	11.92 ± 5.41	-	-
9		007216-56-0	Neo-alloocimene, stab.	16.9818	1136	1131	8.73 ± 1.57	6.92 ± 0.05	-	-
10		013877-91-3	β-Ocimene	12.9660	1037	1037	4.51 ± 0.83	4.05 ± 0.2	-	-
11		034995-77-2	Trans-Linalool oxide	15.1406	1088	1086	-	6.07 ± 0.45	-	-
12		000527-84-4	o-Cymene	12.2771	1021	1022	4.62 ± 3.17	5.17 ± 0.89		-
13		013474-59-4	Trans-α-Bergamotene	26.1710	1438	1435	-	-	8.49 ± 1.97	-
14		000644-30-4	α-Curcumene	27.0580	1473	1483	4.11 ± 2.78	-	13.36 ± 5.02	7.30 ± 2.72
15		030021-74-0	γ-Muurolene	27.2364	1480	1477	-	-	-	2.93 ± 1.48
16		073209-42-4	(+/-)-Trans-Calamanene	28.3895	1527	1529	-	-	-	1.37 ± 0.14
17	fatty acid derivatives	000124-19-6	Nonanal	15.7984	1103	1104	-	-	-	1.15 ± 0.22
18		004440-65-7	3-Hexenal	4.0479	-	-	3.01 ± 0.31	-	-	4.86 ± 0.47
19		006126-50-7	E-4-Hexen-1-ol	5.6801	842	848	0.67 ± 0.30	-	-	1.2 ± 0.09
20		010340-23-5	Cis-3-Nonen-1-ol	17.6725	1155	1156	1.66 ± 0.71	4.31 ± 1.27	-	-
21		000123-29-5	Ethyl nonanoate	22.2212	1294	1296	-	1.17 ± 0.07	-	1.21 ± 0.14
22		000110-42-9	Methyl decanoate	23.1185	1326	1325	2.72 ± 0.31	10.02 ± 2.84	-	2.46 ± 0.58
23		056805-23-3	3,6-Nonadien-1-ol, (E, Z)-	17.7868	1158	1156	0.92 ± 0.17	-	-	-
24	benzene/ phenylpropane compounds	001461-02-5	Dihydrocurcumene	26.4171	1448	1448	-	-	-	1.03 ± 0.17

The total VOC concentration and the vital VOC linalool in each floral structure were calculated, and the results indicated that the labellum, petals, and sepals were the major structures releasing VOCs and linalool. Although possessing the least compounds (6 VOCs), the labellum exhibited the maximum of total VOC concentration and linalool concentration (Fig. 3c), which made it the most fragrant structure of *Phal.* ‘Chanel’ flower. In contrast, the total VOC concentration and linalool concentration in the gynostemium was the lowest (Fig. 3c), rendering its contribution to the floral aromatic profile of *Phal.* ‘Chanel’ negligible. 

### VOC detection of different floral developmental stages

**Fig. 4:**
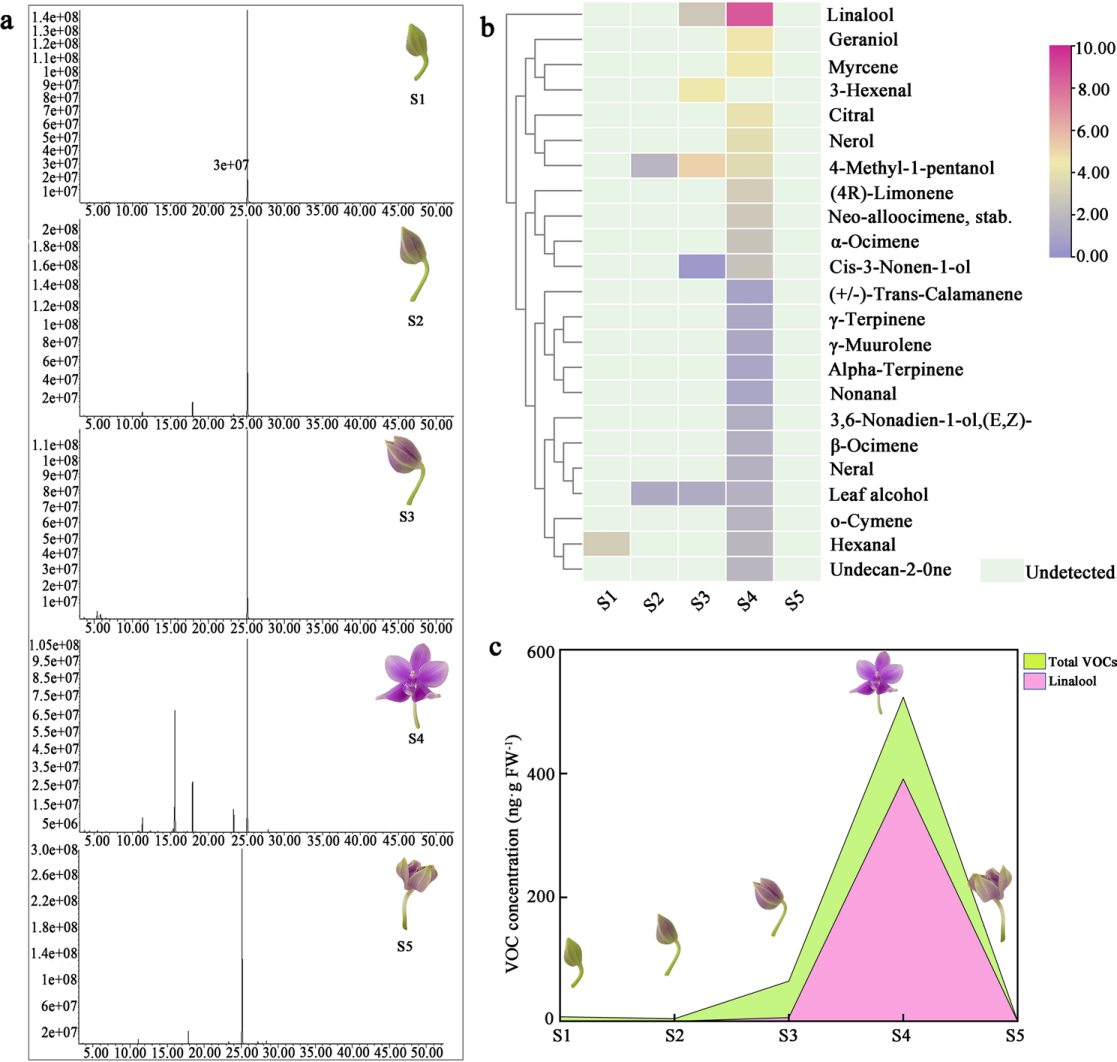
VOC detection and analysis of the five floral developmental stages in *Phalaenopsis* ‘Chanel’ flowers by in vitro extraction. **A** GC-MS total ion chromatograms. **B** Heat map of VOC concentrations. Color bar represents the normalized VOC concentrations, warmer color indicates higher VOC emission, while cool color indicates lower VOC emission. **C** Concentration area map of total VOC and the vital VOC linalool

To investigate scent emission pattern during the flower development in *Phal.* ‘Chanel’, flowers at five developmental stages, from bud to withering (Fig. 1), were used to detect VOCs by HS-SPME/GC-MS (Fig. 4a). Only 1 ~ 5 VOCs were detected in the bud stage S1 ~ S3 (Fig. 4b; Table 4), suggesting that few floral scent was released before blooming. During the bloom stage (S4), the number of VOCs dramatically increased to 22 (Fig. 4b; Table 4). No VOC was detected in the withering stage (S5) (Fig. 4b; Table 4). Both the concentrations of the total VOCs and the vital VOC linalool peaked in the bloom stage (S4) and sharply reduce to zero during withering stage (S5) (Fig. 4c).

**Table 4:** The concentrations of volatile organic compounds (VOCs) in different floral developmental stages of *Phalaenopsis* ‘Chanel’ by in vitro extraction

NO.	Family	CAS	VOC	RT(min)	RI		VOC concentration (ng·g^− 1^)				
					HP-5	NIST	S1	S2	S3	S4	S5
1	Terpene	000078-70-6	Linalool	15.5908	1098	1099	-	-	5.77 ± 1.07	391.05 ± 82.3	-
2		000123-35-3	Myrcene	10.7428	984	991	-	-	-	22.21 ± 0.42	-
3		000106-24-1	Geraniol	21.0311	1256	1255	-	-	-	23.52 ± 6.49	-
4		005392-40-5	Citral	21.5241	1272	1270	-	-	-	16.63 ± 3.78	-
5		000106-25-2	Nerol	20.1978	1229	1228	-	-	-	13.76 ± 0.59	-
6		000106-26-3	Neral	20.5546	1241	1240	-	-	-	2.06 ± 0.37	-
7		000099-86-5	Alpha-Terpinene	11.8292	1011	1017	-	-	-	1.12 ± 0.46	-
8		000527-84-4	o-Cymene	12.2273	1020	1022	-	-	-	2.43 ± 0.4	-
9		005989-27-5	(4R)-Limonene	12.3471	1023	1030	-	-	-	7.07 ± 0.86	-
10		013877-91-3	β-Ocimene	12.8891	1035	1037	-	-	-	1.97 ± 0.6	-
11		000502-99-8	α-Ocimene	13.3731	1047	1047	-	-	-	4.85 ± 0.43	-
12		000099-85-4	γ-Terpinene	13.7722	1056	1060	-	-	-	1.22 ± 0.55	-
13		007216-56-0	Neo-alloocimene, stab.	16.8963	1134	1131	-	-	-	5.82 ± 0.85	-
14		030021-74-0	γ-Muurolene	27.2524	1480	1477	-	-	-	1.22 ± 0.29	-
15		073209-42-4	(+/-)-Trans-Calamanene	28.4500	1529	1529	-	-	-	0.85 ± 0.18	-
16	fatty acid derivatives	000124-19-6	Nonanal	15.6935	1100	1104	-	-	-	1.1 ± 0.07	-
17		004440-65-7	3-Hexenal	3.6390			-	-	22.18 ± 4.7	-	-
18		000066-25-1	Hexanal	3.7266			7.19 ± 1.01	-	-	2.82 ± 0.52	-
19		000928-96-1	Leaf alcohol	5.3682	831	857	-	1.38 ± 0.30	1.46 ± 0.38	2.04 ± 0.93	-
20		000626-89-1	4-Methyl-1-pentanol	5.8334	847	846	-	2.60 ± 0.25	34.85 ± 0.42	12.12 ± 1.28	-
21		010340-23-5	Cis-3-Nonen-1-ol	073209-42-4	1155	1156	-	-	0.3 ± 0.05	4.98 ± 0.65	-
22		000112-12-9	Undecan-2-one	22.2294	1294	1294	-	-	-	2.68 ± 0.93	-
23		056805-23-3	3,6-Nonadien-1-ol, (E, Z)-	17.7812	1158	1156	-	-	-	1.75 ± 0.89	-

During the floral development of *Phal.* ‘Chanel’, both the number and concentrations of VOCs progressively increased from the bud stages (S1 ~ S3) to bloom stage (S4), and peaked in the bloom stage (S4). As a results, the flowers in the bloom stage (S4) possessed the strongest fragrance. After the bloom stage, no VOC was detected in the withering stage (S5), making the withering flowers scentless. As floral scent serving as an important signal substance for attracting pollinators, the changes in floral aromatic profile of *Phal.* ‘Chanel’ with the floral development might be consistent with the pollination biology characteristics of the flower.

### Dynamic monitoring of VOCs in different blooming days

**Fig. 5:**
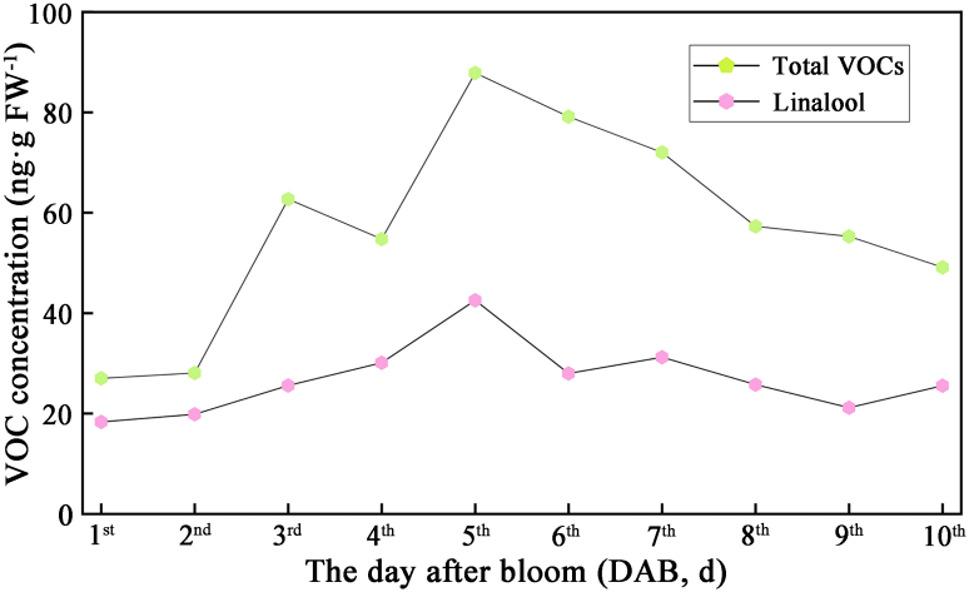
The concentrations of total VOCs and the vital VOC linalool in different blooming days of *Phalaenopsis* ‘Chanel’ flowers by in vivo extraction

*Phalaenopsis* has a long flowering period, and a single flower of *Phal.* ‘Chanel’ can keep blooming for several days. Our previous observations indicated that the perceived scent intensity of *Phal.* ‘Chanel’ flowers varied with the number of bloom days. Therefore, VOCs of the same flower was dynamically monitored by using in vivo adsorption from the 1st day after bloom (DAB) to 10th DAB (Fig. 5; Table 5).

**Table 5:** The concentrations of volatile organic compounds (VOCs) in *Phalaenopsis* ‘Chanel’ flowers from the 1st day after bloom (DAB) to 10th DAB by in vivo extraction

NO.	Family	CAS	VOCs	RT(min)	RI		VOCs Concentration(ng/g)									
					HP-5	NIST	1st DAB	2rd DAB	3rd DAB	4th DAB	5th DAB	6th DAB	7th DAB	8th DAB	9th DAB	10th DAB
1	Terpene	000078-70-6	Linalool	15.60982	1098	1099	18.34 ± 3.93	19.87 ± 4.11	25.59 ± 3.2	30.14 ± 4.29	42.54 ± 1.73	27.99 ± 3.41	31.22 ± 13.33	25.77 ± 1.03	21.17 ± 8.59	25.57 ± 6.37
2		000123-35-3	Myrcene	10.84792	987	991	-	-	3.99 ± 0.04	3.95 ± 0.01	4.05 ± 0.11	3.93 ± 0.02	4.04 ± 0.01	-	3.95 ± 0.07	-
3		000106-24-1	Geraniol	21.02454	1256	1255	-	-	8.37 ± 0.72	-	7.85 ± 1.2	5.77 ± 1.54	5.43 ± 1.53	4.75 ± 0.75	5.33 ± 1.3	4.17 ± 0.27
4		005392-40-5	Citral	21.51584	1271	1270	4.66 ± 0.43	-	8.81 ± 3.71	6.87 ± 2.48	13.27 ± 1.21	9.38 ± 0.35	11.88 ± 4.28	8.02 ± 0.54	10.2 ± 3.16	7.34 ± 1.63
5		005989-27-5	(4R)-Limonene	12.45328	1025	1031	-	-			3.9 ± 0.02	3.92 ± 0.02	-	-	-	-
6		000106-25-2	Nerol	20.18818	1229	1228	-	-	7.37 ± 2.17	5.71 ± 0.91	7.76 ± 0.35	7.09 ± 1.37	7.3 ± 2.07	6.73 ± 1.21	6.44 ± 1.69	-
7		000106-26-3	Neral	20.58946	1242	1240	-	-	4.51 ± 0.23	4.04 ± 0.17	4.42 ± 0.03	4.25 ± 0.18	4.29 ± 0.3	4.1 ± 0.11	4.18 ± 0.28	3.98 ± 0.09
8	fatty acid derivatives	000124-19-6	Nonanal	15.78427	1103	1104	4.01 ± 0.05	4.27 ± 0.21	4.08 ± 0.02	4.07 ± 0.05	4.06 ± 0.11	3.97 ± 0.02	3.98 ± 0.04	4.07 ± 0.05	4.02 ± 0.09	4.18 ± 0.05
9		000123-92-2	Isoamyl acetate	6.39835	866	866	-	-	-	-	-	4.39 ± 0.07	-	-	-	-
10		000539-90-2	Isobutyl butyrate	9.39385	950	955	-	-	-	-	-	4.07 ± 0.06	-	-	-	-
12		000106-27-4	Isoamyl butyrate	13.81580	1057	1056	-	-	-	-	-	4.38 ± 0.13	-	-	-	-
13		000112-31-2	Decanal	19.45969	1205	1206	-	3.93 ± 0.04	-	-	-	-	3.87 ± 0.02	3.86 ± 0.01	-	3.88 ± 0.00

The results showed that the concentrations of total VOCs and the vital VOC linalool increased progressively from 1st DAB to 5th DAB, and peaked on 5th DAB, then gradually declined from 6th DAB to 10th DAB (Fig. 5). The results indicated that the floral scent intensity gradually increased in the early bloom stage, and decreased in the late bloom stage. The flower of *Phal.* ‘Chanel’ on 5th DAB was the strongest scent emission.

### VOC variation with the circadian rhythm

**Fig. 6:**
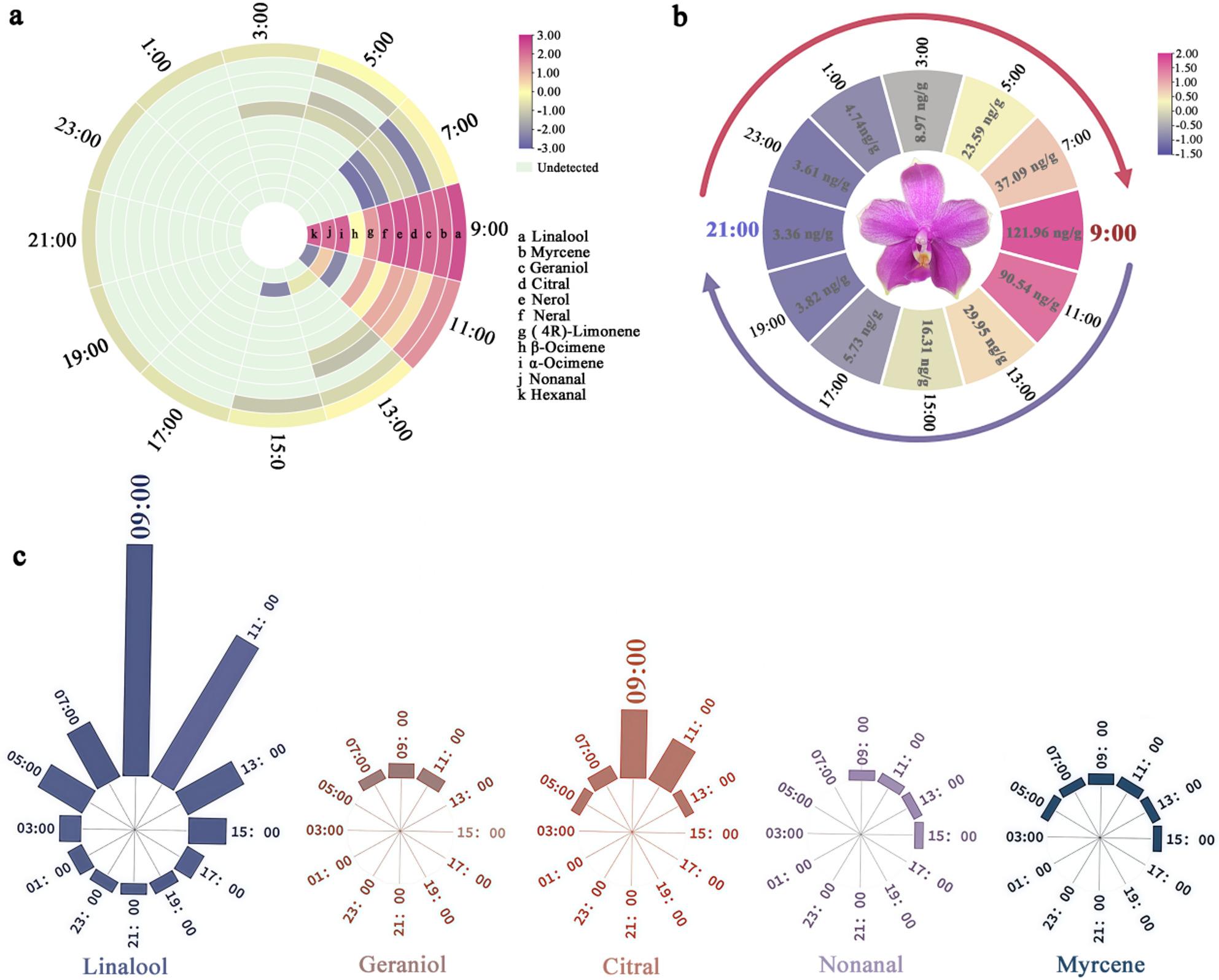
The circadian rhythm of floral scent emission in *Phalaenopsis* ‘Chanel’ by in vivo extraction. **A** Heat map of VOC concentrations. **B** Heat map of total VOC concentration. **C** The concentration variation of the five key VOCs

To explore the circadian rhythm of floral scent emission in *Phal.* ‘Chanel’, VOCs of the same flower on 5th DAB was dynamically monitored in a 24-hour period by using in vivo adsorption. The results showed that the number of VOCs varied with different time in a 24-hour period (Fig. 6a). The greatest diversity of VOCs was detected at 09:00 am, followed by 11:00 am. After 11:00 am, the number of VOCs decreased progressively. The least diversity of VOCs was observed from 17:00 to 1:00, with only one VOC detected (Fig. 6a). From 3:00 to 7:00 at dawn, the number of VOCs increased significantly (Fig. 6a).

The concentration of total VOCs increased from 03:00 and reached the peak value at 09:00 (121.96 ng·g FW^− 1^, Fig. 6b). Although a slight decline was observed at 11:00, total VOCs remained a high concentration at 96.54 ng·g FW^− 1^ (Fig. 6b). Therefore, the flowers exhibited the strongest floral scent between 09:00 and 11:00 in a 24-hour period. The concentration of total VOCs decreased sharply at 13:00, and dropped to the low level from 17:00 to 1:00 (3.36 ~ 5.73 ng·g FW^− 1^, Fig. 6b), during which the floral scent was weak or barely perceptible.

Among the five key VOCs identified in *Phal.* ‘Chanel’, only the vital VOC linalool was consistently detected asross all time points in the 24-hour period (Fig. 6c). The other four key VOCs were detected only during dawn and part of daytime (Fig. 6c). These findings supported the vital role of linalool in shaping the floral aromatic profile of *Phal.* ‘Chanel’. The concentration dynamics of linalool and citral were consistent with that of total VOCs, exhibiting a clear circadian rhythm. Similar to total VOCs, the peak concentrations of linalool and citral were obtained at 9:00 (Fig. 6c), reaching 65.47 ng·g FW^− 1^ and 19.50 ng·g FW^− 1^, respectively (Table 6).

**Table 6:** The concentrations of volatile organic compounds (VOCs) in *Phalaenopsis* ‘Chanel’ flowers in the 24-hour period by in vivo extraction

NO.	Family	CAS	VOCs	RT(min)	RI		VOCs Concentration(ng/g)											
					HP-5	NIST	9:00	11:00	13:00	15:00	17:00	19:00	21:00	23:00	1:00	3:00	5:00	7:00
1	Terpene	000078-70-6	Linalool	15.6057	1098	1099	65.47 ± 3.82	46.88 ± 4.3	18.24 ± 2.12	10.9 ± 0.98	5.73 ± 0.82	3.82 ± 0.31	3.36 ± 0.52	3.61 ± 0.57	4.74 ± 0.97	6.36 ± 0.63	14.75 ± 2.84	17.9 ± 0.53
2		000123-35-3	Myrcene	10.8380	987	991	3.36 ± 0.61	3.22 ± 0.4	2.74 ± 0.23	2.68 ± 0.04	-	-	-	-	-	-	2.69 ± 0.02	2.77 ± 0.25
3		000106-24-1	Geraniol	21.0246	1256	1255	4.41 ± 0.35	3.94 ± 0.59	-	-	-	-	-	-	-	-	-	3.24 ± 0.65
4		005392-40-5	Citral	21.5202	1272	1270	19.5 ± 3.11	13.6 ± 0.12	2.95 ± 0.31	-	-	-	-	-	-	-	3.04 ± 0.13	4.47 ± 0.93
5		000106-25-2	Nerol	20.1966	1229	1228	11.43 ± 1.61	8.38 ± 0.11	3.11 ± 0.39	-	-	-	-	-	-	2.61 ± 0.2	3.11 ± 0.08	3.31 ± 0.33
6		000106-26-3	Neral	20.5924	1242	1240	3.41 ± 0.84	3.08 ± 0.55	-	-	-	-	-	-	-	-	-	2.71 ± 0.28
7		005989-27-5	(4R)-Limonene	12.4115	1024	1031	2.87 ± 0.4	2.86 ± 0.34	-	-	-	-	-	-	-	-	-	2.69 ± 0.06
8		013877-91-3	β-Ocimene	12.9514	1037	1037	2.67 ± 0.29		-	-	-	-	-	-	-	-	-	-
9		000502-99-8	α-Ocimene	13.3991	1047	1047	2.82 ± 0.32	2.74 ± 0.26	-	-	-	-	-	-	-	-	-	-
10	fatty acid derivatives	000124-19-6	Nonanal	15.7863	1103	1104	3.17 ± 0.03	3.03 ± 0.03	2.91 ± 0.02	2.73 ± 0.08	-	-	-	-	-	-	-	-
11		000066-25-1	Hexanal	4.0280	-	-	2.85 ± 0.32	2.81 ± 0.47	-	-	-	-	-	-	-	-	-	-

Overall, the floral scent emission of *Phal.* ‘Chanel’ displayed a distinct circadian rhythm in both the composition and concentration of VOCs. The floral scent intensity was the strongest between 09:00 and 11:00, and became barely perceptible in the evening during a 24-hour period. Given that floral scent serves as an important chemical signal for pollinator attraction, the observed circadian rhythm of floral scent emission in *Phal.* ‘Chanel’ might be related to the pollination biology of the genus *Phalaenopsis*.

## Discussion

Floral scent is an important ornamental trait in plants. The differences in floral aromatic profiles among different plants are mainly due to the differences in the composition and concentrations of VOCs released by flowers. In orchids, the key floral VOCs vary greatly among different genera and species. The key VOC of *Cymbidium goeringii* flowers was farnesol [20]. In *Dendrobium* genus, the key floral VOCs of *D. chrysotoxum* and *D. wardianum* were ethyl linolenate, methyl linoleate [21]. In the present study, the key VOCs identified in *Phal.* ‘Chanel’ were linalool, citral, myrcene, geraniol, and nonanal, among which linalool was the vital VOC determining the floral aromatic profile of *Phal.* ‘Chanel’. Although the key VOCs varied among the native species and cultivars of *Phalaenopsis* [8, 10, 22, 23], linalool is a common key VOC in *Phal.* ‘Nobby’s Pacific Sunset’ [10], *Phal. bellina* and *Phal. violacea* [8, 22].

The present study showed that the floral scent phenotype of *Phal.* ‘Chanel’ is determined more by sensory dominance than by VOC diversity alone. While five compounds were identified as key VOCs (OAVs > 1), monoterpene linalool predominated the aromatic profile with an exceptional OAV of 1443.5, dwarfing the other four components. This result indicated that the sweet and pleasant scent of *Phal.* ‘Chanel’ was primarily dictated by a single potent monoterpene, rather than a synergistic contribution from a diverse array of compounds. This linalool-dominated aromatic profile was consistent with previous findings in scented *Phal.* bellina, *Phal.* violacea, and *Phal.* ‘Nobby’s Pacific Sunset’, where linalool and its derivatives also served as the vital VOCs [1, 8, 10, 22]. However, many cultivars, such as *Phal.* ‘Tzu Chiang, *Phal.* Peter’s Pride and *Phal.* ‘Cherry Tomato’, exhibited the complex scent profiles combining floral, fruity, and herbal scents, which were driven by a diverse array of key VOCs [17].

The floral scent emission varies in different floral structures. For scented flowers, VOCs are mainly emitted from the petals, although pistils, stamens and calyxes also contribute to floral scent in some degree [9, 24]. In the scented *Phal.* ‘Nobby’s Pacific Sunset’, the highest VOC concentrations were detected in the sepals and petals, while the lowest VOC in the gynostemium [10]. In the present study, *Phal.* ‘Chanel’ exhibited a distinct spatial pattern, i.e., the total VOC and the vital VOC linalool concentrations were the highest in the labellum, and lowest in the gynostemium (Fig. 3c). *Phalaenopsis* flowers with the colorful perianths are pollinated by bees [11, 25]. Bees are primarily attracted from a distance to visual stimuli, whereas landing depends upon both visual and olfactory signals [26]. In orchid, labellum is the optimal landing site for pollinator, because the labellum is often enlarged and ornamented with a wart-like structure, providing a holdfast for the insect to grasp with its front legs [27]. In *Phal.* ‘Chanel’, the dazzling yellow spots (Fig. 1) and the strong scent of the labellum might attract bees to land directly on the labellum. The labellum is very close to the anther and stigma, which may facilitate more efficient pollination.

The compositions and concentrations of VOCs released by flowers vary at different developmental stages. Generally, the scent emission increases from the floral bud to blooming flower, then decreases at the withering stage. The blooming flowers with optimum state for pollination, the increased scent emission is beneficial to attract pollinator [28]. This scent emission pattern in the floral developmental stages was also observed in scented *Phal.* ‘bellina’ [8] and *Phal.* ‘Chanel’ studied here. *Phal.* ‘Chanel’ emitted the greatest diversity VOCs and the highest concentrations of the total VOCs and the vital VOC linalool at the bloom stage, and no VOCs at the withering stage. Moreover, the floral scent emission also varies depending on the number of blooming days. While VOCs in *Phal.* bellina became detectable around the 3rd day after bloom (DAB) and peaked on the 5th DAB [2, 8], *Phal.* ‘Chanel’ exhibited an earlier onset of scent emission, with detectable VOCs on the 1st DAB. Despite differences in onset, both *Phal.* ‘Chanel’ and *Phal.* bellina exhibited a synchronized peak in floral scent intensity on the 5th DAB [2, 8]. This finding could provide a clear guideline to obtain samples of *Phalaenopsis* flowers with the strongest scent tensity.

In most scented flowers, the scent emission shows a distinct circadian rhythm, which could be closely associated with different pollination strategies formed during the plant evolution. *Antirrhinum majus* emited methyl benzoate in the daytime to attract diurnal pollinators, such as bees [29]. In the genus *Petunia*, distinct pollination syndromes might be evolved in association with bee-visitation (*P. integrifolia* spp.) or hawk moth-visitation (*P. axillaris* spp.). In order to attract night pollinator hawk moth, *P. axillaris* showed a circadian rhythm of benzenoids with an emission peak at night, which was absent from *P. integrifolia* [12]. In scented *Phal.* ‘Nobby’s Pacific Sunset’, *Phal.* bellina and *Phal.* violacea, the floral VOC emission mainly occurred in the daytime, and the emission peak of *Phal.* ‘Nobby’s Pacific Sunset’ was detected between 9:00 and 13:00 [10, 22]. The floral VOC emission of *Phal.* ‘Chanel’ studied here also presented a circadian rhythm with an emission peak between 9:00 and 11:00. Bees are important pollinators of *Phalaenopsis* [11], and their pollination behaviors are restricted to the daytime, exhibiting a distinct circadian rhythm [30]. We propose that the circadian rhythm of floral scent emission in the scented *Phalaenopsis* might be a coevolutionary adaptation to the diurnal rhythm of the pollinator bees in order to maximize reproductive success.

## Conclusion

In the present study, floral scent emission patterns of *Phal.* ‘Chanel’ were described. A total of 21 VOCs were detected in blooming flowers, among which linalool was identified as the vital VOC determining the sweet and pleasant scent of *Phal.* ‘Chanel’. The scent emission of *Phal.* ‘Chanel’ was multi-dimensionally regulated by spatial distribution and temporal dynamics. The scent emission pattern in spatial distribution was that the highest concentrations of both total VOCs and linalool were localized within the labellum, identifying it as the most fragrant floral structure. In the temporal dynamic pattern, the scent emission varied with floral development and circadian rhythm, and peaked in the bloom stage, especially on the 5th day after bloom (DAB) between 9:00 am ~ 11:00 am. We hypothesize that the pattern of floral scent emission might be a coevolutionary adaptation to the pollinators. By localizing intense olfactory signals in the labellum and synchronizing the scent emission peak with the activity of pollinators, *Phal.* ‘Chanel’ likely optimizes its pollination efficiency and reproductive success.

## Supplementary Information

Supplementary Material 1: Data 1. Standard curve for VOC content caculation of *Phalaenopsis* 'Chanel' flowers through in vivo extraction.

Supplementary Material 2.

## Data Availability

Data is provided within the manuscript. All datasets generated or analyzed during this study are available from the corresponding author upon reasonable request.
