# Supplementary material for: Multidimensional analysis of floral scent emission patterns in Phalaenopsis ‘Chanel’
Source: BMC Plant Biol. 2026 Apr 25;26:993. doi: 10.1186/s12870-026-08738-w (PMC13251233; doi:10.1186/s12870-026-08738-w)
Supplement: Supplementary file 1 — Supplementary Material 1: Data 1. Standard curve for VOC content caculation of Phalaenopsis 'Chanel' flowers through in vivo extraction. [file 12870_2026_8738_MOESM1_ESM.docx]

**Supplementary data1: VOCs of the blank controls in the different extraction methods (in vitro extraction and in vivo extraction)**

**Table 1 VOCs of the blank control for in vitro extraction**

| No. | CAS | VOC | RT (min) | Peak area | | |
| --- | --- | --- | --- | --- | --- | --- |
|  |  |  |  | Control-1 | Control-2 | Control-3 |
| 1 | 000106-42-3 | p-Xylene | 6.1139 | 25226131 | 13149006 | 14704848 |
| 2 | 000629-50-5 | Tridecane | 22.4030 | 9976251 | 8414109 | 7177498 |
| 3 | 076649-16-6 | Ethyl trans-4-decenoate | 24.6741 | 35534473 | 29991590 | 30845086 |
| 4 | 067233-91-4 | Ethyl 9-decenoate | 24.7257 | 9411685 | 9054531 | 9066722 |
| 5 | 000110-38-3 | Decanoic acid, ethyl ester * | 25.1625 | 21010089315 | 20886932123 | 21547753799 |
| 6 | 1000131-95-9 | 4-Decenoic acid, ethyl ester | 25.4915 | 28221865 | 27162854 | 27415862 |

* 1‰ (v/v) ethyl decanoate (CAS 000110-38-3) was used as an internal standard substance.

**Table 2 VOCs of the blank control for in vivo extraction**

| No. | CAS | Library/ID | RT（min） | Peak area | | |
| --- | --- | --- | --- | --- | --- | --- |
|  |  |  |  | Control-1 | Control-2 | Control-3 |
| 1 | 000541-05-9 | Cyclotrisiloxane, hexamethyl- | 4.664 | 10493529 | 7413506 |  |
| 2 | 000112-40-3 | Dodecane | 19.2927 | 25967880 | 14015356 | 12460224 |
| 3 | 000629-50-5 | Tridecane | 22.41103333 | 16182660 | 9855538 | 9822579 |

**
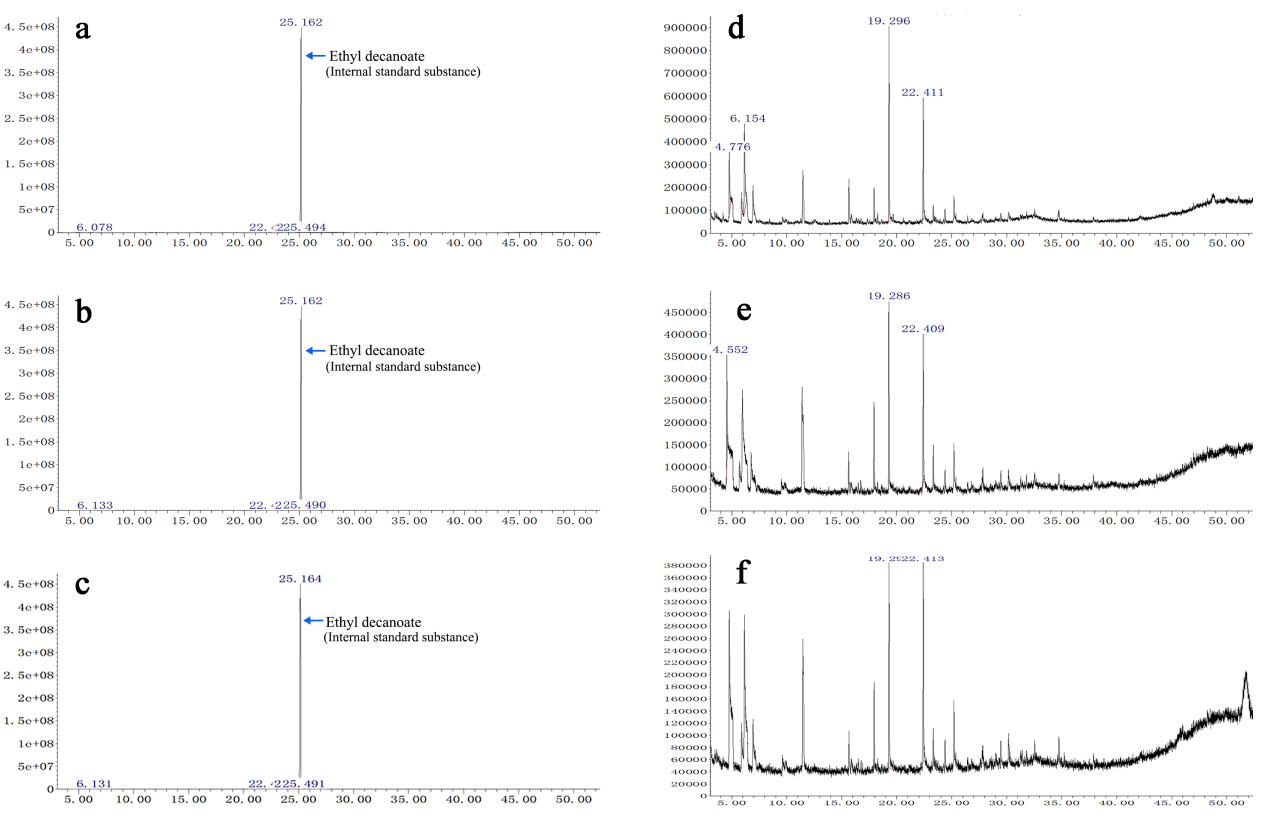
Figure 1 GC-MS total ion chromatograms (TIC) of the blank control for in vitro extraction (a-c.) and in vivo extraction (d-f.)**
