# Supplementary material for: Multidimensional analysis of floral scent emission patterns in Phalaenopsis ‘Chanel’
Source: BMC Plant Biol. 2026 Apr 25;26:993. doi: 10.1186/s12870-026-08738-w (PMC13251233; doi:10.1186/s12870-026-08738-w)
Supplement: Supplementary file 2 — Supplementary Material 2. [file 12870_2026_8738_MOESM2_ESM.docx]

**Supplementary data1: examination of the vital VOC identification, and standard curve for VOC content calculation of *Phalaenopsis* 'Chanel' flowers through in vivo extraction**

1. **Materials and methods**

**1. 1 Examination of the vital VOC identification**

Considering linalool with the highest concentration in *Phal.* 'Chanel', the standard substance of linalool (YuanYe Bio-Technology Co., Ltd., Shanghai, China) was introduced to check VOC identification. The standard substance of linalool was diluted with n-hexane. 1 μl linalool solution was put into 20 ml SPME bottle (Thermo Fisher Scientific, Waltham, MA, USA). Then SPME bottle was promptly capped, and the SPME fiber was inserted into the capped vial. The adsorption of floral VOCs took place at a temperature of 30 ℃ for 30 minutes. Once HS-SPME extraction process was complete, the SPME fiber was transferred to GC-MS injection port for desorption at 250 °C for 1 minute. Subsequently, GC-MS is employed to collect data. The GC-MS parameters were described by Song et al.^[17]^.

In order to check the linalool identification, GC-MS comparison between the floral VOCs of *Phal.* 'Chanel' and the standard substance of linalool was conducted.

**1.2 Standard curve for VOC content calculation of *Phalaenopsis* 'Chanel' flowers**

The standard substance of linalool (YuanYe Bio-Technology Co., Ltd., Shanghai, China) was gradient diluted with n-hexane to 4000, 6000, 60000, 400000, and 600000 -fold, respectively. As a result, the final concentrations of linalool were adjusted to 217.50, 145.00, 14.50, 2.175, and 1.45 ng·μl^-1^. 1 μl linalool solution was put into 20 ml SPME bottle. The HS-SPME extraction and GC-MS analysis were conducted as the description above.

1. **Results**

**2.1 Examination of the linalool identification**

**In the GC-MS analysis, retention time (RT) of linalool in** *Phal.* 'Chanel' flower was consistent with that of the linalool standard substance (Figure S1). Thus, the VOC identification of linalool studied here was confirmed to be correct.

**Figure
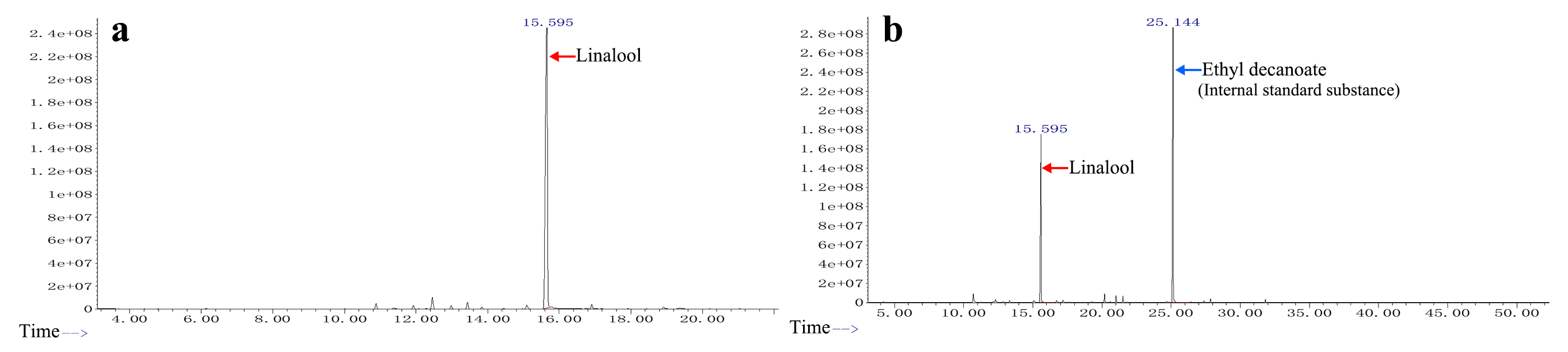
1 GC-MS total ion chromatograms (TIC) of the linalool standard substance and *Phal.* 'Chanel' flower**

a. TIC of the linalool standard substance. b. TIC of *Phal.* 'Chanel' flower.

**2.2 Standard curve**

The peak areas of different linalool contents were collected through GC-MS (Table S1). In order to obtain the standard curve of linalool, a linear regression analysis was conducted between the linalool contents and peak areas. Standard curve was as follows: *y*=4.97×10^7^*x* -1.85×10^8^ (R^2^=0.9992), where *y* represents the peak area and *x* represents the VOC content (ng). For in vivo extraction, the content of each VOC could be caculated by this standard curve.

**Table 1 The peak areas of different linalool contents**

| **Linalool content (ng)** | **Peak area** |
| --- | --- |
| 217.5 | 10720377013 |
| 145 | 6889233655 |
| 14.5 | 356132576 |
| 2.175 | 13801888 |
| 1.45 | 7878953 |


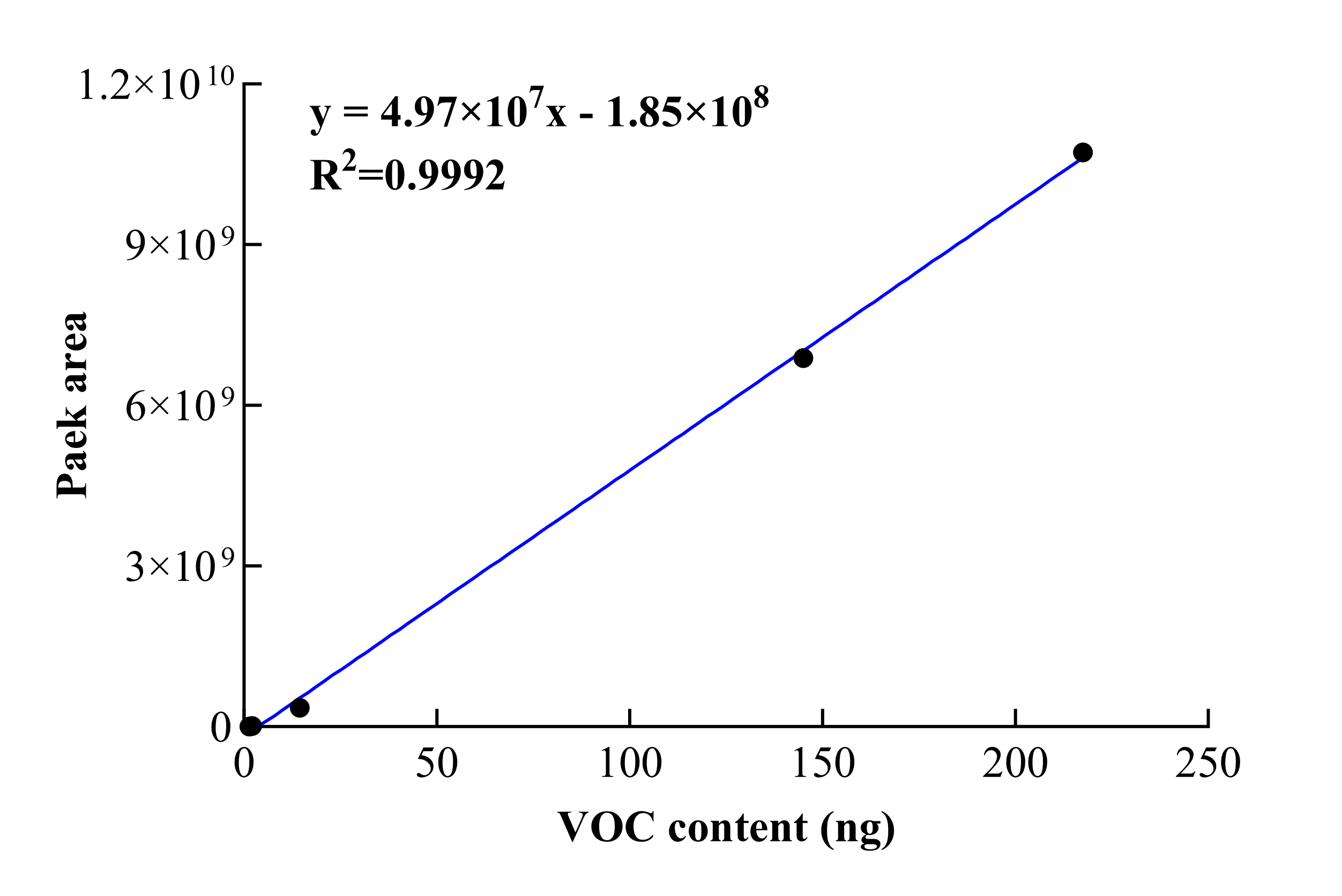
**Figure 2** Standard curve for VOC content calculation of *Phal.* 'Chanel' flowers by in vivo extraction
